# What are patients’ experiences of discontinuing clozapine and how does this impact their views on subsequent treatment?

**DOI:** 10.1186/s12888-023-04851-4

**Published:** 2023-05-22

**Authors:** Jennifer Southern, Phil Elliott, Ian Maidment

**Affiliations:** 1 Aston University, Birmingham, B4 7ET England; 2 Present Address: Cheshire and Wirral Partnership NHS Foundation Trust, Chester, CH2 1BQ England

**Keywords:** Clozapine, Emotions, Shared decision making, Antipsychotic agents, Treatment adherence and compliance

## Abstract

**Background:**

Discontinuing what is considered the most effective treatment for treatment-resistant schizophrenia may precipitate feelings of failure or a relapse of illness. Clozapine treatment is discontinued for a variety of reasons, including non-adherence, intolerance, or lack of efficacy. Patients’ experiences of discontinuing the “best” treatment and the impact on perceptions of subsequent antipsychotic treatment are important in developing an understanding of the factors affecting people’s treatment choices. This study is the first of its type, seeking to explore people’s perspectives on clozapine discontinuation.

**Method:**

Semi-structured interviews with sixteen patients who had received clozapine and discontinued treatment—thirteen males and three females, age range: thirty-two to seventy-eight years old—were audio-recorded and transcribed. A modified inductive approach to analysis, based on grounded theory, was taken to identify commonalities and differences in patients’ perceptions.

**Results:**

The three main themes identified from participants’ experiences were:

1. positive and negative effects of treatment;
2. feelings of agency, being the capacity to make decisions about treatment and act independently;
3. choice of treatment in the future.

Participants exhibited agency in making choices about medication, including risking relapse, while attempting self-management of medication effects. Different participants perceived the same side effect as beneficial or intolerable. Variation in subsequent treatment choices was reported, with some participants favouring depot (long-acting) injections. A participant was frightened when not told about clozapine’s side effects, which led to the participant not being engaged in future treatment decisions. Others, despite suffering serious adverse effects, retained positive perceptions of clozapine; they experienced despair at finding an effective alternative.

**Conclusions:**

Experiences with clozapine discontinuation evoked powerful emotions and resulted in clozapine being the benchmark for other treatments. Knowledge, agency, and being in control were important to participants in relation to treatment. Personal perceptions of treatments or beliefs about illness could lead to non-adherence. People value the clinician listening to their experiences to better understand their perspective, enabling concerns about medication to be addressed through true shared decision making.

**Trial registration:**

NHS Health Research Authority and Health and Care Research Wales, IRAS Project ID 225753, Research Ethics Committee (REC) reference: 18/NW/0413, 25/06/2018.

**Supplementary Information:**

The online version contains supplementary material available at 10.1186/s12888-023-04851-4.

## Introduction

Considered the gold standard medication for treatment-resistant schizophrenia, clozapine [1–3] has numerous side effects that may contribute to discontinuing treatment [2, 4–7]. Discontinuing clozapine may be due to a rare but serious side effect such as myocarditis, neutropenia, seizures, or constipation, or due to several persistent and intolerable effects such as sedation or weight gain [2, 5–7]. Other factors may mean clozapine is discontinued, for example, if it is ineffective or due to personal beliefs about illness [7–12]. There are retrospective case note studies describing the consequences of clozapine discontinuation [4, 6], where the risk of relapse is high and the clinical outcome is usually poor [4, 5]. Clozapine is reserved for treatment-resistant schizophrenia (TRS) [11] and there is no consensus on which treatment to use next [1, 8, 13].

When the “gold standard” antipsychotic [14, 15] is not completely effective or its side effects are intolerable, this may be expected to impact significantly on the person’s view of clozapine and their emotional response to treatment [1, 8, 16–18]. Treatment-resistant schizophrenia is not the only condition in which the negative description of ‘treatment failure’ is used [1, 19–21]. From service user interviews, a study of people with epilepsy describes treatment failure as eliciting “strong feelings of loss of control and vulnerability” [21]. Failure is used frequently in relation to cancer treatments; a small study by Siu et al. (2013) [20] identifies patients experiencing an initial loss of hope with palliative treatment. Feelings about hope, self-blame, and loss of control, are expressed by people living with schizophrenia [1, 10, 22]. The word “failure” and the connotations of the best or last treatment not being successful elicit strong emotions [1, 18]. Seeking the opinion and feelings of the person about being a “treatment failure” is not widely examined in any condition [23–29].

Literature searches of medical databases identified studies mentioning “experience” or “perceptions,” mostly detailing psychiatrists’ views and clinical experience [6, 11, 13, 16, 30–33]. Three studies interviewed people about their experiences of clozapine shared care [33–35], identifying trust and partnership working as important [35]. Angermeyer et al. (2001) [32] interviewed patients, relatives, and clinicians, about clozapine treatment. They concluded that the three groups’ views of clozapine differed significantly [32]. De Silva and Pai (2014) [13] proposed investigating why people discontinue clozapine, the authors were contacted and confirmed the research was never fully commissioned. Information regarding personal experience or perceptions of discontinuing clozapine was not identified in a broad internet search [13, 34, 36–39].

Non-adherence with medicines for long-term conditions, including treatment-resistant schizophrenia, is not unusual and is commonly cited as a reason for discontinuing antipsychotics [7, 22, 28, 31, 40]. Fear of becoming unwell and recurrence of negative experiences are described by people discontinuing antidepressants [41] as well as antipsychotics [32, 35]. Clinicians are advised to identify potential causes of non-adherence and manage side effects [6, 12]; there is less emphasis on understanding the patient perspective, enabling people to participate in shared decision making [40, 42–44] to minimise non-adherence with clozapine. In the main, discontinuation of any type of medicine is examined in terms of clinical measurements and not patient experience [4, 24, 45, 46], making it an important area of research.

People with schizophrenia are at risk of dying twenty years younger than the general population. Using an effective antipsychotic long-term, particularly clozapine, reduces mortality, as described by Tiihonen et al. [47] and Crump et al. [48]. Factors including, side effects, fear of coercion, and a lack of knowledge, insight or trust in clinicians are commonly cited as barriers to physical health improvements and adherence amongst people with schizophrenia [5, 10, 42, 49]. Following periods of non-adherence with antipsychotics, people’s recall of events can be poor, with the associated loss of control [28, 30] or insight, which has implications for shared decision making [40]. People’s perceptions are important in maintaining treatment adherence in this long-term condition [10, 13, 18, 50]. Wellbeing and feeling in control are important for people with schizophrenia [10, 28, 49, 51]. Maintaining treatment with an effective antipsychotic, such as clozapine, is key to controlling symptoms and improving health outcomes overall [17, 52, 53].

Identifying why people discontinue clozapine and the impact this experience has on subsequent treatments may assist in personalising treatment [6–8, 11, 13, 16]. Shared decision making through clinician and patient working in partnership [43, 54], building trust, sharing knowledge, and understanding beliefs or concerns, is believed to lead to better medicine adherence [1, 9, 22, 43, 44, 50, 55–58]. Effectively treating schizophrenia promptly facilitates better management of social and physical health issues for optimal overall health outcomes [10, 59]. Therefore, it is vital to understand the views of patients on treatment discontinuation, particularly clozapine, because it is uniquely effective [17, 52, 53] in treatment-resistant schizophrenia. This exploratory study is a step towards understanding people’s experiences with clozapine, ultimately informing effective shared decisions in treatment-resistant schizophrenia [6–8, 11, 13, 16].

### Aims of the study

The study set out to establish the reason for clozapine being discontinued and explore people’s experiences and perceptions of discontinuing clozapine from the patients’ perspective. The impact those perceptions and experiences had subsequently on views of other treatments was then explored.

## Material and methods

### Design and ethics

This was a qualitative study [60] to elucidate information about participants’ experiences and perceptions of their treatment discontinuation through semi-structured interviews, using a question and topic guide (see Table 1). Approval for the study, which interviewed potentially vulnerable people about their treatment, was obtained from the Health Research Authority/NHS Ethics and Aston University Governance Committee. Consolidated Criteria for Reporting Qualitative Research (COREQ) checklist [61] was followed as per Appendix 1. The interview question and topic guide (Table 1) was prepared with assistance from a psychologist and two patient and public involvement representatives [62]. A report, exported by the clinical pharmacist, from the Clozaril® Patient Monitoring Service (CPMS) database identified patients under the care of the organisation who had discontinued clozapine between 2001 and 2019. The report contains minimal information: the name of the participant, registration/identification number, name of the registered clinician, date of initiation, and date of discontinuation. One-to-one interviews in a familiar environment allowed participants to reveal personal information freely and in a confidential manner.

**Table 1:** Questions and topic guide

**Question**	**Prompts**				
Please could you tell me what you remember about your clozapine treatment?	Can you remember when and why you started on clozapine?	How did you feel while you were taking it, was it for long?	Can you tell me why you stopped it?	How do you remember feeling when you stopped it?	Do you feel the same about the clozapine and stopping it now?
Please could you tell me more about your experience with other antipsychotic treatments since the clozapine was discontinued?	Have you taken any other antipsychotics since taking clozapine, do you know which ones?	Are you taking an antipsychotic now?	Which antipsychotic are you taking?	What do you think about it?	Of the antipsychotics you have stopped can you tell me why they were stopped and how you felt when they stopped?
Have you experienced side effects with antipsychotics? If you have please tell me what happened?	Which antipsychotic do you think caused which side effects?	Which of these side effects, if any, have bothered you the most?	-	-	-
Adherence with medicines is difficult long term for any illness. What happens when you stop taking antipsychotics, do you think you need to take them for the immediate future?	Do you believe antipsychotics are necessary to treat schizophrenia and can you explain why you believe that?	Have you felt any different after stopping antipsychotics, in what way?	Have you tried alternative treatments instead of antipsychotics?	-	-
Thinking about when you stopped clozapine is there anything else which could have helped you when you stopped it or if you could restart clozapine again would you and can you explain your reasons?	If not clozapine which antipsychotic do you think has been the best for you?	Can you explain why that is?	-	-	-

### Invitation to participate

**Fig. 1:**
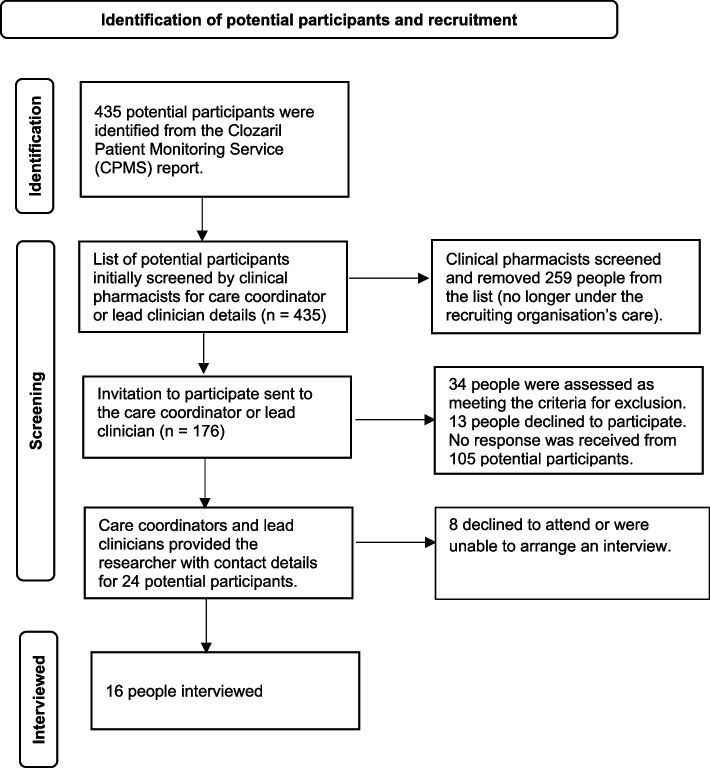
Identification of potential participants and recruitment flow diagram

Potential participants were identified from a large secondary care mental healthcare organisation in North West England, as per the flow diagram in Fig. 1. The population is predominantly white and Caucasian and has a relatively high proportion of older people compared to the rest of the country. Ethnicity data were not formally collected, but from observation, the participants reflected the local population. Geographically, there are large rural areas, but there are also some areas of urbanisation and significant deprivation. The following criteria were applied:

#### Inclusion criteria

- Participants had been registered for Clozaril®, under the care of the health organisation, with a diagnosis of treatment-resistant schizophrenia;
- Participants had taken clozapine for a minimum of two weeks (the usual period for gradually increasing the dose into the therapeutic range);
- Participants’ clozapine treatment had been discontinued.

#### Exclusion criteria

- Those without the capacity to consent to participation in the study were not interviewed;
- Where the care coordinator or lead clinician considered it not to be in the person’s best interest for their mental health, they were not invited for an interview.

A mixture of convenience and purposeful sampling was used. Convenience sampling was employed as all potential participants were approached. This was necessary as the reason for discontinuation was unknown to the researcher until after the interview [60] and consent to access the notes had been obtained. Purposeful sampling was undertaken in so far as interviewing continued until interviews had been conducted for participants with a range of reasons for discontinuation (non-adherence, blood dyscrasias, or side effects) and no new themes emerged [60, 63].

### Interview process and consent

Interviews were conducted between September 2018 and April 2019 and were 20 to 90 min in duration. Where appropriate, for safety or participant assurance, care coordinators or relatives were in attendance for the interview. On two occasions, care coordinators were present, and one participant chose to have a relative present for support. Immediately before conducting the interview, consent was obtained for interview participation and access to medical records and databases. The interviews were audio recorded, transcribed verbatim, and anonymized by the researcher after each interview. Interviews were conducted following the questions and topic guide in Table 1 and undertaken either in the participant’s home or clinical team base, using the participant’s preferred location. Interview data was stored in an anonymized format, as per the ethics approval, on the healthcare trust’s secure IT system until the study was completed. Separate locations were used for downloaded digital audio recordings and the typed Word® transcripts. Data analysis throughout the transcription process allowed adjustment of the interviews to pursue emerging themes and identify when data saturation appeared to have been reached [62, 64, 65].

### Data analysis

This study’s methods had a basis in grounded theory, as defined by Charmaz 2014 [66]; with limited research in this area for comparison, an inductive approach [60, 62] was taken, remaining open to patients’ experiences and perceptions while recognising the researcher’s clinical preconceptions. The researchers carried out thematic analysis [60] using a grounded, inductive approach, generating themes as the interviews were conducted and transcribed. Emerging themes and coding were discussed with both clinical and academic research supervisors throughout the study. No new themes or perspectives on identified themes emerged relating to the research question after interview fourteen. Two further, prearranged, interviews were conducted, with no additional emerging themes. Data saturation was considered to have been reached [60, 63] according to the grounded theory criterion, and data collection ceased [63]. The robustness of the analysis [62, 64] was supported by checking and discussion with the researcher’s supervisors.

Initial coding and revision of the interview process commenced following the first interview and was a continuous process using constant comparison [64]. Open coding [60, 64] was conducted from printed interview transcripts, referencing interview field notes, using a colour-coded scheme. Data immersion was enabled by the researcher transcribing the interview data into Word® documents, which facilitated coding. Data were analysed for repetitions, differing viewpoints, and deviant cases [62] relating to perceptions, experiences, and treatment. Deviant cases were pursued by listening to audio recordings for confirmation of nuances and comparing them with other interview transcripts to identify any further contrasts in experience or expression.

In the next stage of analysis, segments of interviews with the same code were copied into separate documents under the code description. Segments of text could appear under more than one code. All interview segments under the code description were then examined for similarities and differences in perceptions or experiences within the code. Codes were examined in relation to each other, identifying similarities and differences across the codes, to complete axial coding [60, 64]. Further refinement generated three main themes relating to the research question. These themes will be presented with clinical details confirmed from participants’ medical records.

### Reflexivity and validation

The researcher reflected on their role as researcher and clinician, recognising the potential influence of their preconceptions and any previous clinical relationship with study participants [62]. Member checking was not undertaken as the participants’ views were analysed and presented in the context of others; as individual views may be contrary to other perspectives, member checking was considered a less reliable way of validating the data [62, 67]. Dissenting voices were analysed, and opposing views are reported in the results with supporting quotes from interviews [62].

## Results

Medical records and the CPMS database were checked for the sixteen participants to confirm the reason for discontinuation, time on clozapine treatment, age, sex, and diagnosis. The data, which identified that a range of reasons for discontinuation had been included and that participants were eligible to take part in the study, is summarised in Table 2.

**Table 2:** Table of clinical information taken from medical records

**Participant (Pseudonym**^a^**)**	**Age (years)**	**Sex**	**Diagnosis**	**Reason for discontinuation (from medical records)**
Mary	62	Female	Paranoid schizophrenia	Neutropenia ‘red’ result
Rick	52	Male	Paranoid schizophrenia	‘Red’ result. Neuroleptic malignant syndrome (NMS)
Alf	37	Male	Paranoid schizophrenia	Raised eosinophils. Myocarditis not confirmed
Jane	35	Female	Paranoid schizophrenia	Non-adherence, then ‘red’ result
Mark	40	Male	Schizophrenia	Chaotic nature of adherence, to clozapine
Dave	51	Male	Paranoid schizophrenia	Non-adherence
Ben	34	Male	Paranoid schizophrenia	Repeated non-adherence
Ann	52	Female	Paranoid schizophrenia	Non-adherence, missed > 48 h, declined re-titration
Luke	44	Male	Paranoid schizophrenia	Non-adherence
Tom	36	Male	Paranoid schizophrenia	Clozapine causing constipation
Harry	61	Male	Schizophrenia	Severe constipation, faecal impaction (not paralytic ileus)
Bob	78	Male	Paranoid schizophrenia	Non-adherence, abdominal pain and constipation
Keith	55	Male	Schizoaffective disorder	Intensity of side effects
Sid	46	Male	Paranoid schizophrenia	Cardiac problems
James	32	Male	Psychosis	Felt very lethargic with little benefit
Fred	36	Male	Psychosis	Clozapine is 'making him ill', side effects he can’t tolerate

^a^All participants have been given a pseudonym to represent them in the presentation of the results and this is unrelated to their identity

Reasons for discontinuation will now be presented along with the three main themes identified from interviews: (1) positive and negative experiences; (2) feelings of agency; and (3) feelings about future treatment. No correlation was found between age, the reason for discontinuation, and experience with clozapine. Three participants, Fred, James, and Alf, took clozapine for less than two months, but all other participants took it for two or more years. Fred and James felt they could not tolerate clozapine, but Alf had to stop due to a serious adverse effect.

Conventions relating to quotes are available in Table 3.

**Table 3:** Figure legends (conventions and abbreviations used in results)

Convention/abbreviation	Application
Pseudonym	The pseudonym given to each participant is listed in Table 2 and follows any quote. This pseudonym may also be referred to in the analysis
(Round brackets)	Surround the participant’s pseudonym in plain text, following a quote
Text box, italics, and double quotation marks	Indicate direct quotes from participant interviews. There may be more than one related quote contained in each box, and a quote may be referred to elsewhere in the results where it is pertinent to more than one theme
Three full stops…	Indicate where text has been removed from the quote for brevity, not to alter the meaning
[Square brackets]	Contain questions from the researcher for clarification as to what the participant is referring to. For example, a particular medication to which they do not refer by name in that phrase
(anti)*psychotic	The participant used psychotic and antipsychotic in place of each other throughout the interview
QTc	Part of the ECG recording
®	Used where participants referred to medication by brand name. The generic name of the medication is also included in square brackets, except for Clozaril® (clozapine) and Depixol® (flupentixol)

### Positive and negative experiences of treatment

Mary and Rick felt clozapine was the best treatment despite experiencing potentially fatal side effects. Disappointment and self-blame were evident when clozapine, experienced as the most effective antipsychotic, could not be continued:

*“It actually helped me in more ways than anything else had… used to make me happy…felt annoyed with myself because I can’t get back to what I was on Clozaril®…it actually saved me from all the voices and that, and I’m a wreck now…where Clozaril® eased my mind and I slept right through* (Mary)”

*“I feel a bit disappointed… they made me stop it …is the best antipsychotic I’ve been on… I didn’t believe it was the clozapine that made me as ill as I was* (Rick)”

Discontinuing clozapine could have a significant impact, with deterioration in mood and ability to function; some participants, like Mary, had felt dreadful since the discontinuation of clozapine. Conversely, others, like Harry, who perceived clozapine to be ineffective or intolerable, felt relief on discontinuation. Harry was a dissenting voice whose whole experience with clozapine was negative. The experiences were often a mixture of great benefits and significant adverse effects. Keith describes below that he found clozapine both frightening and fantastic; this mirrored the experience of others:

“*Because of the tremendous weight gain I was frightened of having a heart attack…It was absolutely fantastic, it worked 100% against the symptoms* (Keith)”.

“*No didn’t work at all and had severe constipation…It was terrible, I didn’t want to get up in the morning and face life. It made it worse* (Harry)”

Side effects were described by all participants, forming an important part of the participants’ experience of treatment and its discontinuation. Generally, side effects were viewed negatively and as a reason to discontinue. A prime example was weight gain, particularly in association with clozapine. However, there was the opposing view that a little weight gain was beneficial to physical health, with corresponding disappointment about losing weight after discontinuing clozapine:

“*I gained a bit of weight on clozapine…the voices were nearly gone…I was a bit gutted I’ve lost weight* (Mark)”

Differing experiences of sedation with clozapine also demonstrate opposing perceptions of the same side effect. Participants linked improved sleep with improved symptom control and perceived it as restorative, as Mary described above. Equally, some found that oversedation made functioning in daily life a struggle and a powerful factor for clozapine discontinuation:

“*[On clozapine] I remember waking up at one point and me wife had been to hospital and come home and I hadn’t even woke up* (Luke)”

Positive and negative experiences with clozapine were used by participants as personal benchmarks against which to measure experiences with other treatments, as the quotes above from Mary, Harry, and Keith demonstrate.

### Feelings of agency

Perceptions about illness were of not being in control, and this could be a consequence of medicine discontinuation, as described by Tom. Participants could not always identify when they were becoming ill and felt others were in control or knew more about the situation than they did. Participants had little recall of what had occurred when acutely unwell and felt compelled to take antipsychotics due to other people’s opinions. Feeling powerless and coerced, as Bob did, resulted in him declining clozapine tablets:

“*I end up in hospital, I get sectioned … everyone else sees it, I don’t see the change…I just don’t know what’s going on…I don’t remember it properly… [Do you think you need to take the antipsychotics in the immediate future?] Yes, I do I’ve got no choice* (Tom).”

“I didn’t want to take them because they were (anti)*psychotic…just, the nurses at medication time used to come round and give me a handful of tablets to take because they used to say if you just have one or two (anti)*psychotic tablets we can do away with all the others, I just refused them, I was (anti)*psychotic at the time (Bob).”

Agency regarding antipsychotic treatment was evident when antipsychotics were changed or discontinued. When clozapine was abruptly discontinued due to life-threatening effects, participants, such as Rick, felt frightened about relapse and helpless because they did not have agency to choose what they considered the most effective treatment. Previous experience of discontinuing medicines impacted beliefs about what might happen when becoming unwell:

“*The voices got very bad, I got very paranoid…I have hurt myself and I think things like that might happen… to my mind that* [clozapine] *was the best one I’d ever been on so I wanted to try it again* (Rick)”.

Agency could be restricted by clinical reasons, as for Rick, and taken at face value, there is an impression that the doctor makes the decisions about antipsychotic treatment. This was not always the case, as participants, like Luke, discontinued clozapine of their own volition. Limited agency was exercised by refusing clozapine, as Bob did above. Changes to medicines and self-management of side effects were engineered through poor adherence, as demonstrated by Luke below. Luke and others were honest during the interviews about poor adherence. Taking a reduced dose and becoming unwell led to a change in treatment:

“*I didn't take clozapine as regular as I was supposed to and that was making me a bit worse…but as I say the side effects were too aggressive… that’s why I had to come off it* (Luke)”

Doctors discontinued clozapine treatment, but Dave demonstrated subversive agency by hiding some clozapine from the care team and taking it when he thought he needed to:

“*They* [Mental Health team] *stopped me taking it…but I kept some back … I continued taking them, I wasn’t taking it as I should have…I was just taking drugs and…I was just taking one* [clozapine] *so I wasn’t feeling anxious* (Dave)”

In choosing not to resume taking clozapine fully, Dave demonstrated a degree of agency. It could be implied that Dave may have felt pressured during discussions to accept depot injections. Dave articulated knowing he would only take clozapine ad hoc; depot injections were not chosen through coercion but as an assurance to himself:

“*Got it in my head that the depot was better than Clozaril® and so I said I’d go on the depot, they offered for me to go on the Clozaril® and I said no…if I’d stayed on tablets I wouldn’t have taken them… I want to stay on the depot* (Dave)”.

Choosing depot injections felt less restrictive to Jane than the monitoring of oral clozapine medication. Jane exerted agency, utilising a depot to avoid restrictions of a Community Treatment Order (CTO), clozapine not being discontinued for clinical reasons on this occasion. Jane expressed the feeling that life improved by choosing depot but wished that clozapine was available as a depot. Jane considered clozapine more effective but acknowledged she needed depot to maintain adherence:

“*I wanted to come off my CTO. I think the main reason I was on the CTO was so they could supervise meds, Clozaril®, so I thought if I went on the depot there wouldn’t be medicines to supervise …I came off the CTO… I live in a nicer place…I did like it [clozapine]…if…you could have it as a depot…then I would go back on Clozaril®* (Jane)”

Depot medication was not an acceptable treatment for all participants but was prescribed following non-adherence with clozapine. Embarrassment was experienced with depot administration as well as side effects. Not all participants felt agency about their subsequent treatment as, demonstrated by Tom disliking depot as much as the clozapine he had discontinued:

*“It was the embarrassment … to jab me in the bum, I hated it. But that was because I didn’t want to take tablets all the time, I hate taking tablets… [Side effects with the injections?] Yes I’d be shaking* (Tom)”

Tom was by now on different oral medication, but reflection about past treatments led participants to think about future treatment options.

### Feelings about future treatment

Previous experience, particularly if the consequences of discontinuing clozapine had been significant, influenced participants when considering how they felt about subsequent treatment and the future. Some participants found the treatment balance to be in favour of depot injections. With the side effects inherent in those medicines taken into account, they found depot more effective or convenient than clozapine:

“[Thinking about clozapine?] *Yes* [It wasn’t of great benefit?] *No not really no…Depixol…I feel a bit lethargic sometimes, but other than that I don’t get any side effects from it*. (Alf)”

There was a reluctance to risk instability and ill health by changing the antipsychotic. The severity of side effects and whether these could be mitigated, were balanced against how well participants felt on clozapine versus after discontinuation. Reconsidering clozapine discontinuation brought some resignation but also hope that clozapine would be effective and tolerated. Some revealed trust in the doctor; although uncertain about taking clozapine again, James would if the doctor thought it was the best option:

“*If the medicines which I am taking now don’t work I will have to go back on clozapine, because it has been said* [by his doctor] *that this is the best medication on the market… I would go on, I would try* (James)”

When clozapine was discontinued due to a contra-indication, some participants felt hopeless and resigned to being unwell with no effective alternative. They felt no one else could understand their despair. Such were the feelings of mental wellbeing that they wanted clozapine not to be to blame and for there to be another explanation for neutropenia. Despite the risks, participants were wistful about being able to take clozapine again with advice and support from clinicians:

“*I know they have tried all the things…and nobody understands…[Is there anything else which could have helped you when clozapine stopped?] A proper medication* (Mary)”

“*I didn’t believe it was the clozapine that made me as ill as I was…I’d go back on it…I’d definitely give it another go*…*maybe a pharmacist could help with what it’s doing physically* (Rick)”

Experiencing severe side effects scared participants, and treatment was discontinued as perceptions were that the body could not tolerate clozapine. Tom received help with treating constipation and may reconsider clozapine in the future if the side effects can be managed, as he thought it was the best antipsychotic. Lack of information about severe constipation undermined Harry’s trust in the doctors, and he was emphatic that he would not take clozapine again. This frightening experience, in which he did not have agency, still affected Harry, making him reluctant to engage with doctors about future treatment choices:

“*I couldn’t handle the constipation, severe constipation…I was having to do myself an enema…Clozapine was the best definitely…If the constipation could be fixed and I knew it was I would stay on clozapine…it was worth it* (Tom)”

“*I was scared… I had to have an operation, severe constipation… [Laxatives?] Laughs… The doctors said not to have them… [Did you know clozapine could cause constipation?] No no-one told me* (Harry)”

With help from the clinicians, some participants, such as Tom, felt it may be possible to manage clozapine’s side effects, with most looking to the clinician to give advice. Participants speaking directly to clinicians, expressing their opinion, and questioning future treatment options, were less evident:

“*They did involve me and I said it has worked really well before and I’ll be happy to go on it again and they worked on me with compliance and accepting my diagnosis and accepting that I needed meds* (Jane)”.

The implications of these findings about experiences and perceptions of discontinuing clozapine will now be discussed in relation to the literature.

## Discussion

The use and discontinuation of clozapine resulted in a strong emotional response, with participants commonly describing emotions such as fear, trust, failure, and hope. The same emotions of fear, unhappiness, and the feeling that “you might die” were described by participants while taking clozapine and on its discontinuation. Subsequent treatment choices were influenced by the balance of positive and negative experiences with clozapine. People experienced the same side effect differently, resulting in very different perceptions; for example, sleep with clozapine could bring a sense of wellbeing or be a reason for discontinuation. Those who were fearful of the physical health effects of clozapine discontinued it with a sense of relief. Some described clozapine as the “best” antipsychotic, “fantastic,” and “helped in more ways than anything else had.” In these cases, clozapine discontinuation was frightening, with a loss of control over mental health and feelings of hopelessness or failure.

Experiences were influenced by feelings of agency [68], feelings of being in control, and feelings of having choice, or not. Treatment choices incorporated beliefs about illness and the consequences of being unwell, as described in the literature [22, 28, 30, 69]. Some participants worried that when clozapine was discontinued, they would relapse or lose control. Being able to restore or remove either good mental or good physical health [49] through receiving antipsychotics is a powerful tool, that participants wanted to be able to control for themselves. Trust may be placed in clinicians when unwell and agency cannot be fully exerted, as some participants and studies suggest [28, 30, 56]. But not everyone was able to seek their clinician’s help. Lack of insight, as perceived by clinicians, does not preclude agency but can create challenges in reaching a shared decision [28, 40]. One participant chose to take clozapine for his own reasons after clinicians thought it had been discontinued; despite being unwell, he had the agency to keep the clozapine hidden from clinicians in his attempt to self-manage his condition.

Putting control of depot injection administration in the hands of clinicians, after discontinuing clozapine tablets, may seem counter-intuitive to having control or agency. Choosing a depot made some participants feel in control of their mental health by receiving regular treatment in a way they found convenient. This is at odds with some views of depot medication being coercive and reducing agency [28, 55, 70] but was not shared by every participant or all studies [70–72]. Some participants perceived subsequent treatment, including depots, as more effective or tolerable than clozapine, which is contrary to findings elsewhere in the literature [8, 32, 73]. Participants had agency in choosing a depot, minimising the restrictions of a community treatment order, for example, thus demonstrating a different perspective, which is supported by reports that the value of depot medication is underestimated [71, 72, 74].

Without effective treatment the future looked bleak. Some participants expressed feelings of hopelessness or self-blame concerning clozapine discontinuation, feelings that are also referenced in the literature regarding schizophrenia and other long-term conditions [1, 75, 76]. Where the symptom resolution had been good, there was regret, expressed almost as self-blame, when the body could not tolerate the physical effects. Participants trusted doctors to consider every possible alternative, including the risks of clozapine re-challenge [2, 73, 77], otherwise feeling resigned to a less effective treatment. Reliance on clinicians’ expertise for an effective solution left participants feeling vulnerable and uncertain about their future. Hopelessness and despair are apparent when one’s future is reliant on medication [1, 18, 22, 75], the most effective medicine cannot be taken, and any remaining treatment choices are less effective.

Previous studies looking at discontinuation suggest clozapine could be re-initiated in most cases with pro-active side effect management [5, 6]. Participants discontinued treatment early when they did not feel they could tolerate the side effects. Regardless of the severity of the adverse effects, if problems with tolerability could be managed, re-trialling clozapine was considered an option. A sense of agency was apparent where there had been some working in partnership and discussion about treatment; this was valued. Some participants were adamant they would not take clozapine in the future. Not being told about the seriousness of constipation with clozapine was frightening for a participant and affected his ongoing interactions with clinicians. Failing to inform people of side effects or resolve issues affects people’s trust [28, 55, 56] which can result in non-adherence and affect future medicine discussions.

The themes of positive and negative effects, agency, and feelings about future treatment are all linked to true shared decision making, defined by NICE 2021 [54] as “a collaborative process that involves a person and their healthcare professional working together to reach a joint decision about care” and incorporating “the person’s individual preferences, beliefs and values” [78]. Feelings about treatment are influenced by a trusting relationship with clinicians [9, 22, 55, 57, 58] which can be built through shared decision making. Participants reflecting on previous clozapine treatment and balancing this with the risks and benefits of current treatment, demonstrate the potential for sophisticated decision making. Clinicians would be well advised to place trust in patients and work in partnership to reach treatment decisions.

## Limitations

All participants were recruited from the same health organisation, limiting the ethnic diversity of potential participants. Having a small pool of participants meant that only limited patient characteristics could be shared where data was collected to avoid participant identification. The study would benefit from being replicated elsewhere on a larger scale, with more participant characteristics. Correlations could not be made between the demographic data collected and the experience of discontinuation.

Reflection was required about the influence the lead researcher’s clinical role may have had on both the researcher and interview participants. At the time of the interviews, the lead researcher was not involved in the clinical care of the participants. The research supervisors, advising on interpretation, had no clinical role, limiting the impact of any influence.

The data requires triangulation with the views of clinicians and participants’ family or friends. In two instances, care team staff were present, but they did not participate in the interviews. When requested by the participant, one relative prompted the participant about events. The researcher maintained focus on her interaction with the participant and their response. The responses from the family member were not included in the analysis as they were only applicable to one participant. However, the role of family and friends in shared decision making is recognised [79] and it would be valuable to incorporate their view into future research of this nature.

## Conclusions

Powerful emotions, including fear, hope, and trust, were expressed about clozapine and its discontinuation. Being scared, through a lack of knowledge about the effects of clozapine, was detrimental to treatment and relationships with clinicians; the power of educating people about their medicines should not be underestimated.

Beliefs about illness and personal perceptions about treatment could result in non-adherence. Open and honest discussion allows for agency in treatment decisions and provides alternative ways to manage medication without resorting to non-adherence. Clinicians must place trust in people to take clozapine regularly and build patients’ trust by addressing fears through shared decision making [44].

Feelings of control relating to illness and treatment were widely expressed as important to people. Even when unwell, people may still demonstrate some agency and trust in clinicians when truly involved in making choices about treatment. Agency was displayed in treatment decisions, with people choosing to have depot injections instead of clozapine, which clinicians may assume to be a less favourable option. This demonstrates the importance of involving people in treatment decisions and for clinicians to be open to unexpected choices [43].

### Future research

This study needs to be replicated in other geographical areas, expanding on the effects of non-adherence, and side effect management. There is value in triangulation with perspectives from clinicians, family, and friends to further explore shared decision making in treatment-resistant schizophrenia.

Understanding the different perspectives is essential to developing an effective approach to implementing the recommendations of previous studies [1, 2, 5–7, 14, 15, 17, 22, 28, 55, 77] about side effect management. This could be achieved by following the Medical Research Council (MRC) guidance [80] to develop and evaluate a complex intervention to support those faced with clozapine discontinuation. A complex intervention would involve supporting clinicians and patients in more than one scenario, requiring more than one type of intervention. A set of interventions could support: i) those who have to discontinue clozapine due to a contra-indication; ii) prevention of discontinuation, including managing adherence; and iii) those who ultimately choose to discontinue clozapine to minimise the risk of relapse.

## Supplementary Information

**Additional file 1:** 

## Data Availability

The qualitative data generated from this research project is not suitable for sharing as per ethical approval and the study protocol.
