# Supplementary material for: What are patients’ experiences of discontinuing clozapine and how does this impact their views on subsequent treatment?
Source: BMC Psychiatry. 2023 May 22;23:353. doi: 10.1186/s12888-023-04851-4 (PMC10204301; doi:10.1186/s12888-023-04851-4)
Supplement: Supplementary file 1 — Additional file 1: [file 12888_2023_4851_MOESM1_ESM.docx]

**Appendix 1 - COREQ Checklist** ^1^

**Domain 1: Research Team and Reflexivity**

**1. Which author/s conducted the interview or the focus group?** The interviews were conducted by Jennifer Southern (JS). Phil Elliott (PE) and Ian Maidment (IM) provided supervision.

**2. What were the researchers’ credentials?** JS and IM are experienced mental health pharmacists. JS is undertaking this project as a research MSc with Aston University. PE & IM are experienced academic and NHS researchers and hold PhDs. IM has led three qualitative projects funded by NIHR Pharmacy UK and supervised many student projects.

**3. What was their occupation at the time of the study?** JS is a senior mental health pharmacist employed at Cheshire and Wirral Partnership NHS Foundation Trust. IM is a Senior Lecturer in Clinical Pharmacy, Aston University. PE is a Senior NHS Research Facilitator at Cheshire and Wirral Partnership NHS Foundation Trust.

**4. Was the researcher male or female?** JS is female, IM and PE are male.

**5. What experience or training did the researcher have?** JS is experienced in care of people living with schizophrenia and has worked in clozapine clinics. PE & IM are experienced academic and NHS researchers and hold PhDs. IM and PE are experienced in mentoring those undertaking research. IM has led three qualitative projects funded by NIHR Pharmacy UK and supervised many student projects.

**6. Was a relationship established prior to study commencement?** JS had met some of the participants in her professional capacity prior to the commencement of the study. JS formally introduced herself as the researcher, clarifying this role and the topic before obtaining consent and commencing the interview.

**7. What did the participants know about the researcher?** The participants knew that JS was a pharmacist at Cheshire and Wirral Partnership NHS Foundation Trust undertaking this study as a researcher with Aston University. They also knew that JS had support from the Trust research team (from PE) and Aston University (from IM).

**8. What characteristics were reported about the interviewer/facilitator?** JS reported that she was an experienced pharmacist and researcher for the study.

**Domain 2: Study Design**

**9. What methodological orientation was stated to underpin the study?** An exploratory, qualitative study was conducted employing semi-structured interviews. Data were analysed using an inductive approach, acknowledging potential for JS to have clinical preconceptions, using thematic analysis within a grounded theory framework.

**10. How were participants selected?** From a report run from the Mylan Clozaril® Patient Monitoring Service of patients from Cheshire and Wirral Partnership NHS Foundation Trust who had discontinued clozapine since 2001, provided they were still under the care of Cheshire and Wirral Partnership NHS Foundation Trust. Clinical pharmacists identified those potential participants still under the care of the NHS trust before providing a list of potential participants and their lead clinician and/or care coordinator to the researcher (JS).

**11. How were participants approached?** The researcher (JS) sent letters of invitation to Lead Clinicians and Care Coordinators asking them to invite potential participants to be interviewed for the study, provided they met the study criteria:

A Participant Information Sheet explaining the study which was included with the letter to be shared with the potential participants. JS visited Community Mental Health Teams and made contacted by phone and email to explain the study and recruitment process. The study was explained at locality clinician and management meetings to raise awareness of the study.

**12. How many participants were in the study?** The final sample comprised of sixteen participants

**13. How many people refused to participate or dropped out?** Twenty one.

**14. Where was the data collected?** The interviews were carried out at community mental health team clinic bases, in the participant’s home accompanied by their Community Psychiatric Nurse or at the participant’s nursing home.

**15. Was anyone else present besides the participants and researchers?** Three participants had their Community Psychiatric Nurse (CPN) present for the interview, the CPNs were observers and did not take an active role in the interview. One participant requested a close relative be present, to whom the participant often deferred. Efforts were made by the researcher to direct interactions to the participant rather than the relative.

**16. What are the important characteristics of the sample?** People were eligible if they had received clozapine for the treatment of schizophrenia for a minimum of two weeks, had stopped treatment and were able to consent to the interview. All were recruited from the same mental health NHS trust. Ethnicity data was not collected but no one was excluded due to a language barrier for example.

**17. Were questions, prompts, guides provided by the authors?** **Was it pilot tested?** The interview schedule consisted of open-ended questions to avoid leading participants’ responses. Prompts were built into the schedule and employed if required. JS developed the interview schedule with input from a psychologist and patient involvement representatives. The interview was piloted with a non-participant volunteer. Permission to record comments made after the recorder was switched off was obtained. Those with questions about their current care were directed to consult their usual care team.

**18. Were repeat interviews carried out?** No

**19. Did the research use audio or visual recording to collect the data?** A digital recorder was used to record the interviews. Data were transcribed verbatim by JS into Word documents.

**20. Were field notes made during and/or after the interview or focus group?** Field notes were made after the interviews.

**21. What was the duration of the interviews or focus group?** The interviews were not formally timed or recorded. From the digital recorder the interviews not including the introduction to the study were twenty to sixty minutes in length.

**22. Was data saturation discussed?** Data saturation was reached and discussed by JS with IM and PE.

**23. Were transcripts returned to participants for comment and/or correction?** Transcripts were not returned to the participants but permission had been obtained to contact participants if the recording was not clear at the point of transcription. Participants were asked if they wished to receive a summary of the research report when the study was concluded.

**Domain 3: Analysis and Findings**

**24. How many data coders coded the data?** JS reviewed and coded the transcripts. JS discussed her findings with IM and PE. JS analysed the data to identify the similarities and differences between the interview transcripts and to develop a set of themes which represent the whole data set. JS discussed her approach to the analysis and themes identified with IM and PE.

**25. Did authors provide a description of the coding tree?** No.

**26. Were themes identified in advance or derived from the data?** Themes were derived from the data. Three key themes were elicited from the data with subthemes within these three themes.

**27. What software, if applicable, was used to manage the data?** Dragon software was used for some of the transcription. Interviews were transcribed into Word documents. The data was manipulated within Word documents, a software package was not used for management or coding of the data.

**28. Did participants provide feedback on the findings?** Participants did not provide feedback.

**29. Were participant quotations presented to illustrate the themes/ findings? Was each quotation identified?** Participant quotes from the interview transcripts are presented in text boxes in the results section. Each quotation is identified, anonymity was maintained and each participant was given a pseudonym.

**30. Was there consistency between the data presented and the findings?** Yes there was consistency between the data presented and the findings.

**31. Were major themes clearly presented in the findings?** The three themes are presented with representative quotations which are discussed in the results section.

**32. Is there a description of diverse cases or discussion of minor themes?** Yes, diverse cases have been discussed and minor themes are also discussed where pertinent to the research.

**Reference**

1. Tong A, Sainsbury P, Craig J. Consolidated criterio for reporting qualitative research (COREQ): a 32- item checklist for interviews and focus group. *Int J Qual Heal Care*. 2007;19(6):349-357. doi:10.1093/intqhc/mzm042
